# Association between desire thinking and problematic social media use among a sample of Lebanese adults: The indirect effect of suppression and impulsivity

**DOI:** 10.1371/journal.pone.0277884

**Published:** 2022-11-28

**Authors:** Emmanuelle Awad, Myriam El Khoury-Malhame, Ecem Yakin, Venise Hanna, Diana Malaeb, Souheil Hallit, Sahar Obeid

**Affiliations:** 1 Social and Education Sciences Department, School of Arts and Sciences, Lebanese American University, Beirut, Lebanon; 2 Centre d’Études et de Recherches en Psychopathologie et Psychologie de la Santé, Université de Toulouse, UT2J, Toulouse, France; 3 School of Pharmacy, Lebanese International University, Beirut, Lebanon; 4 College of Pharmacy, Gulf Medical University, Ajman, United Arab Emirates; 5 School of Medicine and Medical Sciences, Holy Spirit University of Kaslik, Jounieh, Lebanon; 6 Research Department, Psychiatric Hospital of the Cross, Jal Eddib, Lebanon; 7 Applied Science Research Center, Applied Science Private University, Amman, Jordan; Chiang Mai University, THAILAND

## Abstract

**Background:**

Desire thinking, impulsivity and suppression are psychological variables that are intricately related to behavioral addictions. Bearing in mind the scarcity of data on desire thinking, impulsivity, thought suppression and pathological social media use in developing countries such as Lebanon, with the existing literature suggesting a heightened mental health burden associated with this problematic social media use, it becomes all the more important to elucidate their relationship. Our study aims at investigating the association between desire thinking and problematic social media use specifically, and to further test the effect of impulsivity and thought suppression in mediating the relation between the two distinct facets of desire thinking and problematic social media use.

**Methods:**

A cross-sectional study was carried out between November 2021 and March 2022 using a sample of 414 community-dwelling participants aged above 18 years from all Lebanese districts. The data was collected through an online questionnaire including a section about sociodemographic information, the Desire Thinking Questionnaire (DTQ), Impulsive Behavior Scale (S-UPPS-P), White Bear Suppression Inventory (WBSI) and Social Media Disorder Short Form (SMD). The tests used in the bivariate analysis to assess correlates of SMD were the Student t test to compare two means and the Pearson test to correlate two continuous scores. The PROCESS SPSS Macro version 3.4, model four was used to conduct the mediation analysis.

**Results:**

Desire thinking was shown to correlate with increased social media use. Moreover, we found that suppression and lack of premeditation mediated the association between verbal perseveration and social media use disorder whereas suppression and urgency mediated the association between imaginal prefiguration and social media use disorder.

**Conclusion:**

This study provides new insight on a topic of increasing public health concern. Although understudied to date, suppression and impulsivity differentially mediate the influence of both facets of desire thinking on problematic social media use disorder. The current findings point to the highly pervasive issue of social media use disorder and the need to investigate underlying psychological factors that aggravate it to better profile and support individuals struggling with it.

## Introduction

Desire thinking is a cognitive process originally intended to control intrusive experiences related to cravings but ultimately escalates it by voluntarily elaborating a desired behavior on its two dimensions: imaginal prefiguration and verbal perseveration [1]. The first aspect consists of sustaining attention to the information related to desired behavior, generating a tendency to anticipate multi-sensory positive memories whereas the latter includes prolonged self-talks about positive reasons to engage in achievements related to desired behavior. The metacognitive theory [2] stipulates that this trait-like thought process is preserved by beliefs centered around elaborating the desired behavior yet not acting upon it, which increases the craving to go through with addictive behaviors [3].

Desire thinking was first shown to predict various substance addictions such as smoking [4], and alcohol consumption [5]. A recent meta-analysis further documented similar results in behavioral addiction including gambling and sex addiction [6]. Although initially intended to better control negative experiences associated with addictions such as cravings and negative affect, desire thinking may manifest as a maladaptive coping strategy eventually increasing vulnerability to addictions. This is particularly relevant in the case of Internet related maladaptive behaviors such as gaming, pornography and problematic social media use, with an exponential increase in reported numbers of such maladaptive behaviors in the recent years [7]. Desire thinking has been investigated as a predictor of Internet addiction [8] and maladaptive use of social media with platform such as Facebook, Twitter or Instagram [9]. Therefore, better understanding the relationship between desire thinking and such pathological behaviors could facilitate its subsequent exploration as a potential target for the treatment of pervasive behavioral addictions, notably the escalating pathological use of social media worldwide by billions of users.

Other psychological constructs that are closely linked to behavioral and Internet addictions include cognitive and emotional variables such as thought suppression [10] and impulsivity [11]. Higher impulsivity and an inability to regulate one’s behaviors is indeed linked to higher levels of addiction [12]. Similarly, attempting to inhibit or suppress one’s thought increases risk of addiction [13]. This could be the case as thought suppression could be viewed an attempt to ignore unwanted thoughts, which backfires [14]. On the other side, impulsivity is the tendency to react in a fast and incautious manner regardless of consequent repercussions to the self and others [15], increasing addictive tendencies. Impulsivity was further shown to include multiple facets [16], such as negative urgency, lack of perseverance, lack of premeditation, sensation seeking and positive urgency [17], which could reinforce addictions differently. Negative urgency consists of the inclination to behave in an impulsive way when experiencing negative affect [18]. Lack of perseverance is failing to maintain attention and complete challenging tasks [18]. Lack of premeditation is the tendency to act without assessing the consequences of the behavior [18]. Sensation seeking is pursuing certain actions that are pleasurable and enticing despite it being threatening [18].

Impulsivity and thought suppression were consistently studied in correlation to desire thinking [19]. It is important to note that those factors were identified in many different pathways related to cravings and behavioral addictions. For instance, some studies showed that those who engage in attempts to control and suppress addiction-related thoughts had higher desire thinking scores and ended up having increased addictive behaviors [20]. This was found to be significant in relation to problematic use of social media, where desired thinking was defined as a predictor in one study [21] and a mediator in the relation between craving and problematic social media use in another [22]. One recent study showed that impulsivity was a direct predictor of addiction to social media in a sample of adults [23]. Alongside thought suppression, impulsivity was also shown to have an indirect effect on the problematic use of social media [22]. In yet another contemporary research, suppression and impulsivity both mediated the association between thought processes and multiple behavioral addictions, including problematic use of social media [24].

The latest COVID pandemic has particularly inflated the already problematic use of social media platforms [25], as reports of hours spent on social media skyrocketed. Social media addiction has been reported as a pervasive problem in modern societies and raises significant concerns related to mental and physical health [26]. In this context, the Middle East is known to have a high level of Internet accessibility and subsequent smartphone and social media use. Lebanon is one of the Middle Eastern countries with the fastest increase in internet use. In fact in 2017, 80% of Lebanese adults had a smartphone with internet access, in comparison with only 57% in 2015 [27]. Additionally, 72% of Lebanese adults in 2017 reported using social media in comparison to only 53% of French adults and 20% of Indian adults [28]. As such, we decided to investigate the association between problematic social media use and desire thinking in Lebanon. Knowing that desire thinking, impulsivity and thought suppression might intervene at various steps in the etiology of this behavioral addictions [21], we further examined the effect of different facets of impulsivity and thought suppression in mediating the relation between the two distinct dimensions of desire thinking and problematic social media use among a sample of Lebanese adults.

To the best of our knowledge, this is the first time such factorial triads are investigated. Bearing in mind the scarcity of data on desire thinking, impulsivity, thought suppression and pathological social media use in developing countries such as Lebanon, with the existing literature suggesting a heightened mental health burden associated with this problematic social media use [29], it becomes all the more important to investigate the relationships at hand. As such, we hypothesized that higher desire thinking would be correlated with higher pathological social media use, mediated by higher suppression and impulsivity. In addition, the mediation role of thought suppression between desire thinking and problematic social media use was assessed.

## Methods

### Study design and participants

A cross-sectional study, enrolling 414 participants, was carried out between November 2021 and March 2022, using a sample of community-dwelling Lebanese residents aged above 18 years. Due to the social restrictions caused by the COVID-19 pandemic, we used an anonymous, self-administered questionnaire created on Google forms. The link was shared among the participants and sent to all districts/governorates of Lebanon (Beirut, Mount Lebanon, North Lebanon, South Lebanon, and Bekaa) through social networks, using the snowball technique.

### Minimal sample size calculation

A minimal sample of 410 was deemed necessary using the formula suggested by Fritz and MacKinnon [30] to estimate the sample size: $n=\frac{L}{f2}+k+1$, where f = 0.14 for small effect size, L = 7.85 for an α error of 5% and power β = 80%, and k = 8 variables to be entered in the model.

### Ethics approval and consent to participate

The Psychiatric Hospital of the Cross Ethics and Research Committee approved this study protocol (HPC-041-2021). A written informed consent was considered obtained from each participant when submitting the online form. All methods were performed in accordance with the relevant guidelines and regulations.

### Questionnaire and variables

A self-administered, anonymous questionnaire with closed-ended questions was circulated to participants. It required approximately 25–30 minutes to be completed.

### Sociodemographic characteristics

The first part included socio-demographic characteristics: age, gender, educational level, and household crowding index (the latter was calculated by dividing the number of persons living in the house by the number of rooms, excluding the kitchen and the bathroom [31]). The physical activity index was calculated by multiplying the intensity by the frequency by the time of physical activity [32]. Body Mass Index (BMI) was calculated from self-reported height and weight. Participants were asked to rate their financial burden using one question on a scale from 1 to 10, with 10 referring to overwhelming pressure.

### Desire Thinking Questionnaire (DTQ)

It is a self-reported measure of desire thinking that includes 5 items evaluating imaginal prefiguration (e.g. “I imagine myself doing the desired activity”) and 5 others evaluating verbal perseveration (e.g. “If I did not practice the desired activity for a long time, I would think about it continuously”) [33]. All 10 items are rated on a 4-point Likert scale ranging from 1 (never) to 4 (always). Higher scores on the scale indicate higher tendency for desire thinking. The Cronbach alpha values in this study were 0.97 for verbal perseveration and 0.99 for imaginal prefiguration.

### White Bear Suppression Inventory (WBSI)

It is a self-reported measure of thought suppression including 15 items such as “There are things I prefer not to think about” [34]. All items are rated on a 5-point Likert scale ranging from 1 (Strongly disagree) to 5 (Strongly agree). Scores range between 15 and 75 with higher scores on the scale indicate higher tendency for suppressing one’s thoughts. The Cronbach’s alpha in this study was 0.94.

### Social Media Disorder Short Form (SMD)

Validated in Lebanon [35], it is a self-reported measure of problematic social media use including 9 items such as “During the past year have you often felt bad when you could not use social media?” [36]. All items have dichotomous (yes/no) answers. Higher scores on the scale indicate higher tendency for problematic social media use. The Cronbach’s alpha in this study was 0.85.

### I-8 scale

Impulsivity was measured using the I-8 scale, which yields four dimensions: urgency, lack of premeditation, lack of perseverance and sensation seeking on a 6-point scale from 0 (doesn’t apply at all) to 5 (applies completely). Higher scores indicate higher impulsivity level in each of the four subscales: urgency (Cronbach’s alpha = 0.86), lack of premeditation (Cronbach’s alpha = 0.93), lack of perseverance (Cronbach’s alpha = 0.72) and sensation seeking (Cronbach’s alpha = 0.97). The factor structure of the Arabic version of the scale has been previously verified [37].

### Translation procedure

A bilingual translator was responsible for the translation from English to Arabic. A backward translation was then performed by a native English-speaking translator, fluent in Arabic and unfamiliar with the concepts of the scales. Discrepancies were resolved by consensus between translators and researchers.

### Statistical analysis

To check the psychometric properties of the DTQ and WBSI scales, we used an EFA-to-CFA strategy [38]. To ensure adequate sample sizes for both EFA and CFA, we split the main sample using the SPSS computer-generated random technique; there were no significant differences between the two subsamples in terms of mean age, t(412) = .286, p = .775, BMI, t(412) = 1.059, p = .290, and household crowding index, t(412) = .175, p = .861, as well as the distribution of women and men, χ2(1) = .137, p = .711, single and married participants χ2(1) = .590, p = .443 and secondary or less and university level of education χ2(1) = .023, p = .881.

### Exploratory factor analysis

To explore the factor structure of both scales, we computed a principal-axis EFA with the first split-half subsample using the SPSS software [39]. We verified all requirements related to item-communality, average item correlations, and item-total correlations [40]. The Kaiser-Meyer-Olkin (KMO) measure of sampling adequacy (which should ideally be ≥ .80) and Bartlett’s test of sphericity, which should be significant, ensured the adequacy of our sample [41]. The procedure followed for determining the number of dimensions was the Parallel Analysis (PA) [42], using the Pearson correlation matrix. Weighted Root Mean Square Residual (WRMR) were also calculated to assess the model fit (values < 1 have been recommended to represent good fit) [43]. Item retention was based on the recommendation that items with “fair” loadings and above (i.e., ≥ .40) and with low inter-item correlations (suggestive of low item redundancy) as indicated by the anti-image correlation matrix should be retained [44].

### Confirmatory factor analysis

We used data from the second split-half to conduct a CFA using the SPSS AMOS v.26 software. A previous study suggested that the minimum sample size to conduct a confirmatory factor analysis ranges from 3 to 20 times the number of the scale’s variables [45]. Therefore, we assumed a minimum sample of 150 participants needed to have enough statistical power based on a ratio of 10 participants per one item of the scale, which was exceeded in the second subsample. Parameter estimates were obtained using the maximum likelihood method and fit indices. Evidence of convergent validity was assessed in this subsample using the Fornell-Larcker criterion u8, with average variance extracted (AVE) values of ≥ .50 considered adequate [46]. The normed model chi-square (χ^2^/df), the Steiger-Lind root mean square error of approximation (RMSEA), the Tucker-Lewis Index (TLI) and the comparative fit index (CFI). Values ≤ 5 for χ^2^/df, and ≤ .08 for RMSEA, and .90 for CFI and TLI indicate good fit of the model to the data [47, 48].

There was no missing data since all questions were required in the Google form link. The social media use disorder score was considered normally distributed (skewness and kurtosis values between -2 and +2) [49]. The SPSS software v.22 was used to conduct the bivariate analysis of factors associated with SMD; the Student t test was used to compare two means (i.e. sex, marital status and education level), whereas the Pearson correlation test was used to correlate SMD with other continuous scores (impulsivity, desire thinking, etc.). The PROCESS SPSS Macro version 3.4, model four [50] was used to calculate all pathways (Pathway A from the independent variable to the mediator, Pathway B from the mediator to the dependent variable and Pathway C from the independent to the dependent variable). Pathway AB calculated the indirect effect; the latter was deemed significant when the macro generated bias-corrected bootstrapped 95% confidence intervals (CI) did not pass by zero [50]. The covariates that were included in the mediation model were age, body mass index, household crowding index, physical activity (variables that showed a p<0.25 in the bivariate analysis).

## Results

### Sociodemographic and other characteristics of participants

A total of 414 non-clinical participants were included in this study. They had a mean age of 38.58 ± 18.01 years, and 51.2% of them were females. Additional sample characteristics are summarized in Table 1.

**Table 1:** Sociodemographic and other characteristics of the participants (N = 414).

Variable	N (%)
**Sex**	
Male	202 (48.8%)
Female	212 (51.2%)
**Marital status**	
Single	242 (58.5%)
Married	172 (41.5%)
**Education level**	
Secondary or less	133 (32.1%)
University	281 (67.9%)
	**Mean ± SD**
**Age (in years)**	38.58 ± 18.01
**Body Mass Index (kg/m** ^**2**^ **)**	23.91 ± 3.83
**Physical activity index**	25.47 ± 28.44
**Household crowding index (persons/room)**	0.88 ± 0.38
**Financial burden**	5.77 ± 2.14
**Verbal perseveration**	14.40 ± 5.25
**Imaginal prefiguration**	12.78 ± 5.96
**Social media use disorder**	3.41 ± 2.59
**Urgency**	4.97 ± 2.24
**Lack of premeditation**	4.99 ± 2.71
**Lack of perseverance**	5.80 ± 2.39
**Sensation seeking**	6.78 ± 2.81
**Suppression**	60.52 ± 11.78

### Prevalence of social media use disorder

The results showed that 162 participants (39.1%) had social media use disorder as they scored above the cut-off for the pathology.

### Factor analysis

#### DTQ scale

Bartlett’s test of sphericity, χ^2^(45) = 2450.3, p < .001, and KMO (.923) indicated that the DTQ items had adequate common variance for factor analysis. The results of the EFA revealed a two-factor solution, which explained 91.53% of the common variance (item-factor loadings ≥ .80). The WRMR value was also adequate (= .047; 95% CI .032-.058), indicating good fit of the model.

CFA of the two-factor model obtained in the EFA indicated a good fit: χ^2^/df = 165.78/34 = 4.88, RMSEA = .141 (90% CI .120, .162), CFI = .966, and TLI = .955. The convergent validity for this model was adequate, as AVE = 1.06.

#### WBSI scale

Bartlett’s test of sphericity, χ^2^(105) = 2431.1, p < .001, and KMO (.929) indicated that the WBSI items had adequate common variance for factor analysis. The results of the EFA revealed a two-factor solution, which explained 89.51% of the common variance (item-factor loadings ≥ .80). The WRMR value was also adequate (= .013; 95% CI .010-.015), indicating good fit of the model.

CFA of the two-factor model obtained in the EFA indicated a modest fit: χ^2^/df = 667.70/90 = 7.42, RMSEA = .181 (90% CI .168, .194), CFI = .873, and TLI = .852. When adding a correlation between items 2–10, 3–4 and 9–13, the fit indices improved as follows: χ2/df = 466.38/87 = 5.36, RMSEA = .149 (90% CI = .136, .163), CFI = .917, and TLI = .900. The convergent validity for this model was adequate, as AVE = .92. The standardized estimates of factor loadings of both scales were all adequate (Table 2).

**Table 2:** Items of the DTQ and WBSI in English and factor loadings derived from the Exploratory Factor Analyses (EFA) with women and men in the first split-half subsample, and standardized estimates of factor loadings from the Confirmatory Factor Analysis (CFA) in the second split-half subsample.

Item	EFA		CFA	
	Factor 1	Factor 2	Factor 1	Factor 2
**Model 1: DTQ scale**				
1		.93		.96
2		.92		.97
3		.94		.92
4		.92		.93
5		.84		.95
6	.92		.95	
7	.97		.98	
8	.95		.99	
9	.94		.98	
10	.89		.97	
**Model 2: WBSI scale**				
1	.92		.91	
2		.97		.95
3	.95		.91	
4	.94		.88	
5	.97		.86	
6	.94		.94	
7		.97		.98
8	.95		.93	
9	.94		.83	
10	.95		.90	
11	.93		.91	
12		.97		.97
13	.95		.93	
14	.96		.90	
15	.91		.77	

#### Bivariate analysis

The bivariate analysis results are shown in Tables 3 and 4. Older age (r = -0.19) was significantly associated with less social media use disorder, whereas higher verbal perseveration (r = 0.24), imaginal prefiguration (r = 0.21), urgency (r = 0.28), lack of premeditation (r = 0.15), lack of perseverance (r = 0.20) and suppression (r = 0.20) were significantly associated with more social media use disorder.

**Table 3:** Bivariate analysis of the categorical variables associated with social media use disorder score.

Variable	Social media use disorder	
	Mean ± SD	*p*
**Sex**		0.509
Male	3.33 ± 2.66	
Female	3.50 ± 2.53	
**Marital status**		0.616
Single	3.47 ± 2.61	
Married	3.34 ± 2.57	
**Education level**		0.374
Secondary or less	3.24 ± 2.81	
University	3.49 ± 2.48	

Numbers in bold indicate significant p-values.

**Table 4:** Correlation of continuous variables associated with social media use disorder.

Variable	Social media use disorder	
	r	*p*
Age (in years)	-0.19	**<0.001**
Body Mass Index (kg/m^2^)	0.07	0.159
Physical activity index	0.11	**0.027**
Household crowding index (persons/room)	0.08	0.129
Financial burden	0.04	0.425
Verbal perseveration	0.24	**<0.001**
Imaginal prefiguration	0.21	**<0.001**
Urgency	0.28	**<0.001**
Lack of premeditation	0.15	**0.003**
Lack of perseverance	0.20	**<0.001**
Sensation seeking	-0.07	0.141
Suppression	0.20	**<0.001**

Numbers in bold indicate significant p-values; r = Pearson correlation coefficient.

### Mediation analysis

**Fig 1:**
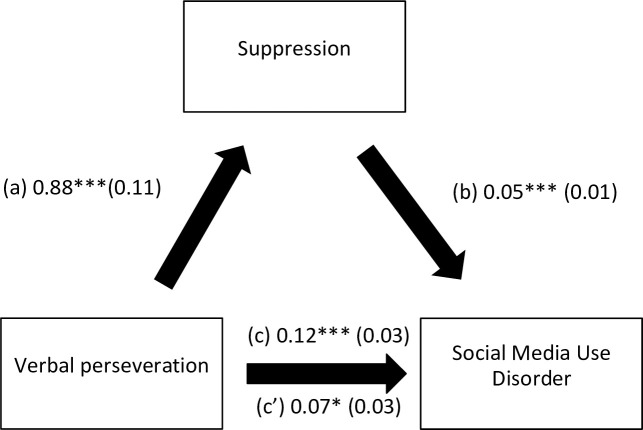
(a) Relation between verbal perseveration and suppression (R^2^ = 20.38%); (b) Relation between suppression and social media use disorder (R^2^ = 13.92%); (c) Total effect of the relation between verbal perseveration and social media use disorder (R^2^ = 9.55%); (c’) Direct effect of the relation between verbal perseveration and social media use disorder. Numbers are displayed as regression coefficients (standard error). ***p<0.001; *p<0.05.

**Fig 2:**
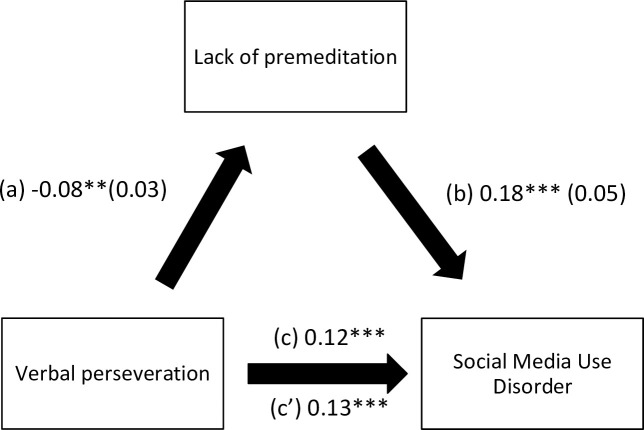
(a) Relation between verbal perseveration and lack of premeditation (R^2^ = 20.38%); (b) Relation between lack of premeditation and social media use disorder (R^2^ = 13.92%); (c) Total effect of the relation between verbal perseveration and social media use disorder (R^2^ = 9.55%); (c’) Direct effect of the relation between verbal perseveration and social media use disorder. Numbers are displayed as regression coefficients (standard error). ***p<0.001; *p<0.05.

**Fig 3:**
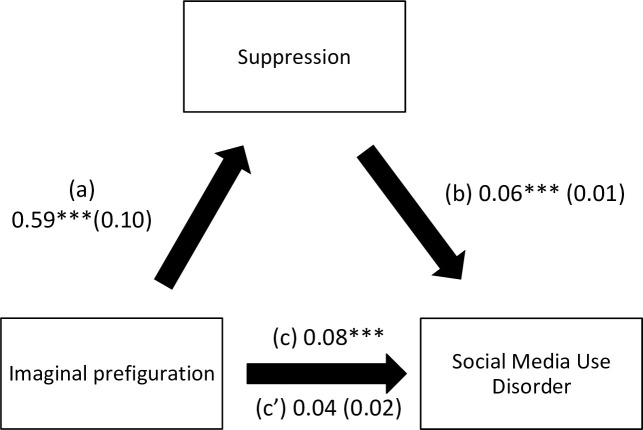
(a) Relation between imaginal prefiguration and suppression (R^2^ = 20.38%); (b) Relation between suppression and social media use disorder (R^2^ = 13.92%); (c) Total effect of the relation between imaginal prefiguration and social media use disorder (R^2^ = 9.55%); (c’) Direct effect of the relation between imaginal prefiguration and social media use disorder. Numbers are displayed as regression coefficients (standard error). ***p<0.001.

**Fig 4:**
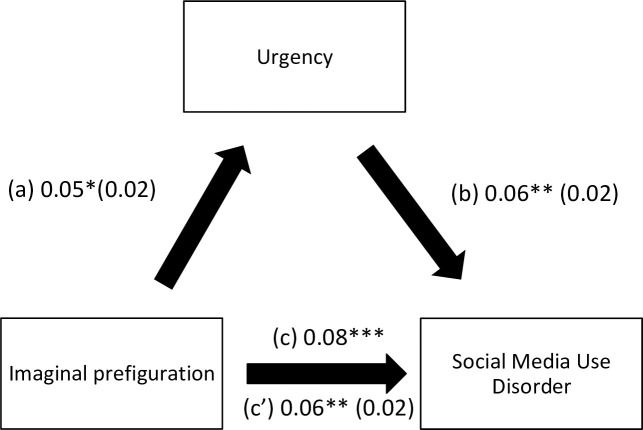
(a) Relation between imaginal prefiguration and urgency (R^2^ = 20.38%); (b) Relation between urgency and social media use disorder (R^2^ = 13.92%); (c) Total effect of the relation between imaginal prefiguration and social media use disorder (R^2^ = 9.55%); (c’) Direct effect of the relation between imaginal prefiguration and social media use disorder. Numbers are displayed as regression coefficients (standard error). ***p<0.001.

The results of the mediation analysis are summarized in Table 5. Suppression (Fig 1) and lack of premeditation (Fig 2) mediated the association between verbal perseveration and social media use disorder. Suppression (Fig 3) and urgency (Fig 4) mediated the association between imaginal prefiguration and social media use disorder. More verbal perseveration was significantly associated with more suppression and more social media disorder, whereas more suppression was significantly associated with more social media disorder. More imaginal prefiguration was significantly associated with more suppression, which was associated with higher social media disorder. More verbal perseveration was significantly associated with less lack of premeditation and more social media disorder, whereas more lack of premeditation was significantly associated with more social media disorder. Finally, more imaginal prefiguration was significantly associated with more urgency and more social media disorder, with higher urgency being significantly associated with more social media disorder.

**Table 5:** Mediation analyses results, taking verbal perseveration / imaginal prefiguration as the independent variables, the impulsivity subscales and the suppression score as mediators and social media use disorder score as the dependent variable.

	Direct effect			Indirect effect		
	Beta	SE	*P*	Beta	Boot SE	Boot CI
**Independent variable: verbal perseveration**						
Suppression	0.07	0.03	0.01	0.05	0.01	0.03–0.07*
Urgency	0.11	0.03	<0.001	0.01	0.01	-0.004–0.03
Lack of premeditation	0.13	0.03	<0.001	-0.01	0.01	-0.03- -0.003*
Lack of perseverance	0.13	0.03	<0.001	-0.01	0.01	-0.02–0.01
Sensation seeking	0.12	0.03	<0.001	-0.001	0.003	-0.01–0.01
**Independent variable: Imaginal prefiguration**						
Suppression	0.04	0.02	0.061	0.03	0.01	0.02–0.05*
Urgency	0.06	0.02	0.006	0.02	0.01	0.003–0.03*
Lack of premeditation	0.08	0.02	<0.001	0.001	0.004	-0.01–0.01
Lack of perseverance	0.07	0.02	0.001	0.004	0.01	-0.01–0.02
Sensation seeking	0.07	0.02	0.001	0.004	0.003	-0.01–0.01

* indicates significant mediation. Covariates: age, body mass index, household crowding index, physical activity.

## Discussion

### Prevalence

Our study investigated the correlation between desire thinking and problematic social media use, and further illustrated differential mediation models of impulsivity facets and thought suppression on the relation between the two dimensions of desire thinking and social use in Lebanese adults.

Our results indicate that 40% of the current sample fit the criteria for pathological social media use. These results are in line with a meta-analysis assessing social media addiction, suggesting that in collectivist countries, such as Lebanon, 31% of individuals are addicted to social media [51]. In neighboring Gulf countries, almost 50% of surveyed people reported perceiving themselves as addicted to social media [52]. Results in Saudi Arabia, another Arab country, indicate an overall rate of 48% of Internet addictions [53], whereas the rate in the Philippines is approximately 25% [54].

### Desire thinking and problematic social media use

As hypothesized, our results show that desire thinking was significantly correlated with problematic social media use, which is similar to previous findings documenting that desire thinking plays a significant role in enhancing addictive behaviors including problematic social media use [55]. Furthermore, desire thinking was not only associated with problematic social media use but is also thought to be an underlying key factor connecting other psychological variables with problematic social media [22]. As such, desire thinking was integrated into a more structured model focusing on behavioral disorders related to the Internet such as problematic social media use disorder and was found to directly influencing addiction and cravings [7].

### Mediation of suppression and lack of premeditation between verbal perseveration and problematic social media use

We found that suppression and lack of premeditation moderated the relation between verbal perseveration and problematic social media use. Previous studies have focused on desire thinking as a moderator variable [22], overlooking differential investigation of its two dimensions and further highlighting the complexity and interdependence of various constructs in determining behavioral addictions. The verbal perseveration dimension of desire thinking indeed consists of self-talk in order to convince oneself to engage in a desirable activity [3]. Verbal perseveration was found to significantly predict a variety of addictive behavior including problematic social media use [21]. Most importantly, it was found to expedite the pathway from normal use of the Internet to addictive use [56], however, it failed to show a direct path to craving online activities, which is known to pave the way to subsequent social media addiction [57]. This suggests the possibility of an indirect pathway between desire thinking, notably verbal perseveration and problematic social media use. Lack of premeditation consists of disregarding the consequences of one’s actions, in addition to intentionally yet inadequately suppressing and ignoring one’s unwanted thoughts that would moderate the backlash of the verbal perseveration aspect of focusing on an unwanted arousing behavior, ultimately reinforcing problematic social media use.

### Mediation of suppression and urgency between imaginal prefiguration and problematic social media use

In the current study, suppression and urgency mediated the association between imaginal prefiguration and problematic social media use. The urgency facet of impulsivity was already found to be a prominent predictor of social media addiction [58]. Similarly, urgency was evidenced as a predictor of smartphone addiction, which was positively correlated with high social media use [59]. Within the same context, higher suppression was associated with increased and more severe problematic smartphone use [60]. Although significant, the contribution of thought suppression to the model is very modest and within range of previous work, and might suggest an alternative route where suppression influences addictive behavior. Desire thinking, specifically imaginal prefiguration, was significantly related to problematic social media use [55]. It could be that imagining the positive results of a desired behavior elicits arousing emotions, in turn triggering a sense of urgency that ultimately increases the focus on the behavioral addiction.

### Thought suppression, impulsivity and problematic social media

Our results indicate that higher suppression was significantly associated with higher social media disorder. First, research has shown that thought suppression has an ironic rebound effect where higher cognitive engagement in suppressing thoughts results in higher probability of engaging in that behavior [61]. Such could be the case that supports the current findings: the higher the thought suppression relating to social media, the higher the possibility of eventually engaging in problematic social media behavior.

Also, two sub-factors of impulsivity, lack of premeditation and higher urgency, had positive relationships with social media disorder. This is consistent with a recent study that showed a positive association between impulsivity and a social media platform, Facebook [62]. Similar results were found in another study where higher impulsivity correlated to higher social media addiction [63]. A breakdown of all subsets of impulsivity further evidenced that all were connected with higher problematic social media use [23]. Moreover, intervention programs targeting lack of premeditation specifically successfully alleviated behavioral addictions overall [64], such as social media use disorder. These connections could be explained by the fact that impulsivity facets were shown to share neural pathways with behavioral addictions [65], with common neurocircuitry for addiction and urgency recently elucidated [66].

### Recommendations

At a time when social media is becoming a seemingly essential element for the daily lives of many people, and whilst reposts documenting its problematic use are exponentially rising, the current results have important implications to better optimize its use. First, it could help profile vulnerable adults whose social media use could become problematic. Second, it could help clinicians better target problematic social media use by employing interventions targeting maladaptive coping strategies in desire thinking, thought suppression and impulsivity. Lastly, taken together, these factors would inform policies to address this rising public health threat. It is indeed noteworthy in that regard that awareness campaigns and behavioral interventions effectively decreased the negative effects of problematic social media use, such as the life skills enhancement program [67].

### Limitations

This study has some limitations. The data was collected through an online questionnaire, which introduces respondent bias. Plus, the snowball technique followed to collect the data predisposes us to a selection bias. Additionally, the Desire Thinking Questionnaire and WBSI are not yet validated in Lebanon; the psychometric properties of those scales in the current study revealed modest results, therefore, findings should be interpreted with caution. Some variables had a low direct effect on social media use disorder. Although the White Bear Suppression Inventory is old, however it is consistently used in recent literature and had a very high Cronbach’s alpha in the current study. The analyses conducted reflect correlational relationships between variables and therefore do not infer causation. Lastly, the lack of active governmental literacy in Lebanon on such novel psychopathological variables makes it difficult to promote effective policies that can be applied in various sectors exposed to excessive social media use including industrial, educational and public health domains.

## Conclusion

This study provides new insight on factors associated with problematic social media use, including desire thinking, impulsivity and thought suppression. This is especially relevant to the Lebanese context, with a population faced with multiple crises, including a global pandemic and a severe socio-economic recession, exposing people to extreme chronic stress. It adds to the literature of a global modern public health issue that is massively present in the daily lives of Lebanese people. The association between desire thinking and problematic social media use disorder being mediated by impulsivity and suppression offers potential targets for future scientific investigations.

### Future studies

The current findings show the pervasiveness of social media use disorder and the need to further investigate factors contributing to it. Although cognitive modalities such as desire thinking and thought suppression are misleadingly defined as inhibiting unwanted thoughts with the goal of controlling unwanted behaviors, this study adds to the panoply of evidence showing an inverse effect of exacerbating maladaptive social media use. Impulsivity, notably with its lack of premeditation and sense of urgency also mediated the association between desire thinking and problematic social media use. These differential factors could be selectively targeted in various interventions to diminish the deleterious mental health burden of behavioral addictions.
